# Micronuclei and Nuclear Abnormalities in Oral Mucosa as Indicators of Genotoxicity in Healthcare Professionals

**DOI:** 10.3390/toxics14010061

**Published:** 2026-01-08

**Authors:** Juana Sánchez-Alarcón, Stefano Bonassi, Mirta Milić, Ninfa Ramírez-Durán, Keila Isaac-Olivé, Rafael Valencia-Quintana

**Affiliations:** 1 Programa de Doctorado en Ciencias de la Salud, Facultad de Ciencias de la Conducta, Universidad Autónoma del Estado de México, Toluca 50180, Estado de México, Mexico; juana.sanchez@uatx.mx; 2 Cuerpo Académico Ambiente y Genética UATLX-CA-223, Laboratorio “Rafael Villalobos-Pietrini” de Toxicología Genómica y Química Ambiental, Facultad de Agrobiología, Universidad Autónoma de Tlaxcala, Ixtacuixtla 90120, Tlaxcala, Mexico; 3 Department of Human Sciences and Quality of Life Promotion, San Raffaele University, 00166 Rome, Italy; 4 Unit of Clinical and Molecular Epidemiology IRCCS San Raffaele Pisana, 00166 Rome, Italy; 5 Division of Toxicology, Institute for Medical Research and Occupational Health, 10000 Zagreb, Croatia; mmilic@imi.hr; 6 Laboratorio de Microbiología Médica y Ambiental, Facultad de Medicina, Universidad Autónoma del Estado de México, Toluca 50180, Estado de México, Mexico; nramirezd@uaemex.mx; 7 Laboratorio de Investigación en Teranóstica, Facultad de Medicina, Universidad Autónoma del Estado de México, Toluca 50180, Estado de México, Mexico; kisaaco@uaemex.mx

**Keywords:** biomonitoring, exfoliated cells, multivariate analysis, occupational exposure, oral cytome

## Abstract

The buccal micronucleus cytome assay (BMCyt) is a validated, non-invasive biomonitoring method used to detect early genotoxic and cytotoxic changes linked to environmental and occupational exposures. Healthcare workers, especially nurses and dentists, are routinely exposed to genotoxic agents such as anesthetic gases, cytotoxic drugs, ionizing radiation, and heavy metals. This study compared seven cytological biomarkers in exfoliated buccal cells from female nurses, dentists, and teachers to assess multivariate cytogenetic differences and potential occupational influences. Samples were collected from 32 nurses, 41 dentists, and 47 teachers, and 3000 cells per participant were evaluated for micronuclei (MN) and six additional nuclear abnormalities. Group differences were examined using MANOVA and permutation MANOVA, followed by pairwise tests, and visualized with Principal Component Analysis (PCA). Significant multivariate differences were found between nurses and both dentists and teachers (*p* = 0.003), supported by permutation tests, while dentists and teachers did not differ. PCA explained 56% of the variance and showed apparent clustering of nurses. Chromatin condensation and MN were the main contributors to group separation. Nurses had significantly higher MN (*p* ≤ 0.001) and karyorrhexis (*p* ≤ 0.0004) than dentist and teachers. Overall, nurses showed a distinct cytogenetic profile consistent with greater genotoxic susceptibility.

## 1. Introduction

The buccal micronucleus cytome assay (BMCyt) is a minimally invasive, well-validated cytogenetic biomonitoring technique designed to assess genotoxicity, cytotoxicity, and cellular proliferation by analysing exfoliated oral mucosal epithelial cells [1,2,3,4]. This assay has been extensively used to evaluate the biological impact of environmental and occupational exposure to genotoxic agents, as well as to investigate the influence of nutritional status, lifestyle factors, and their combined effects on DNA integrity [1,3,5,6].

The presence of micronuclei (MN) and other nuclear abnormalities (NAs) in exfoliated buccal mucosal cells serves as a sensitive biomarker of chromosomal damage, mitotic disturbances, and cytotoxic effects. Their frequency can be modulated by multiple variables such as age, disease status, and behavioural or nutritional habits [7,8]. Increased MN frequencies are consistently associated with exposure to known genotoxic agents, including those classified as carcinogenic by the International Agency for Research on Cancer (IARC) [9]. The BMCyt assay is a practical, cost-effective tool for biomonitoring healthcare workers. By quantifying the frequency of MN and various nuclear anomalies indicative of apoptosis, necrosis, and mitotic disruption, including binucleated cells (BN), karyorrhectic (KR), karyolitic (KL), condensed chromatin (CC), pyknotic (PYK), lobulated nucleus (LN), nuclear bud (NB) cells, the assay provides a comprehensive picture of cellular health and genomic stability [1,10,11]. Therefore, the BMCyt assay is considered a reliable biomarker system for detecting early genotoxic effects and assessing genomic instability in exposed populations [1,3,12,13,14].

In the healthcare field, professionals, such as nurses and dentists, are routinely exposed to a wide variety of hazardous substances that can compromise their short- and long-term health and may exert genotoxic or carcinogenic effects, such as anesthetic gases, cytotoxic drugs, ionising radiation, and heavy metals [15]. Chronic exposure to these agents, often at low but cumulative doses, may result in DNA damage, oxidative stress, and long-term health effects, including increased cancer risk [8,16]. Of particular concern is the potential for mercury exposure, which remains present in some dental materials and clinical procedures and is well recognised for its neurotoxic and cytotoxic effects [17]. This underscores the need for cytogenetic biomonitoring strategies to assess occupational exposure and implement preventive measures to reduce long-term health risks [18]. Nurses and dentists are particularly vulnerable due to the diversity of chemicals used in hospitals and dental clinics, the handling of cytostatic and anesthetic compounds, and the historical use of mercury-containing devices such as thermometers and sphygmomanometers [19,20].

Emerging evidence indicates that chronic occupational exposure to complex mixtures of poorly characterised chemicals, such as anesthetic gases, disinfectants, and heavy metals (mercury), may contribute to DNA damage, genomic instability and cytotoxic effects [16]. Furthermore, individual susceptibility to DNA damage can be exacerbated or influenced by factors such as nutritional deficiencies, metabolic polymorphisms, and psychosocial stress, which may modulate the biological response to toxic exposures [3,8].

In a clinical setting, particularly in dentistry and nursing, healthcare professionals may be chronically exposed to various chemical agents with significant toxicological risks. In dentistry, the preparation of amalgams and the use of anesthetic gases in environments that often lack adequate ventilation and occupational safeguards, along with factors such as lighting conditions and prolonged exposure time in contaminated areas, may further increase the risk of mercury exposure [15,21]. Similarly, nurses may be exposed to cytotoxic drugs, anesthetic gases, ionising radiation, and heavy metals, depending on their work context, including mercury, which has historically been present in thermometers and sphygmomanometers [14]. In some developing countries where these devices are still allowed, records from a single hospital indicate the use of more than 3200 thermometers, 85% of which contained mercury; 624 units were reported broken, releasing approximately 0.646 kg of mercury annually [22]. Additional sources of mercury exposure include tracheostomy tubes and other medical devices [23,24]. Although mercury remains a concern due to its neurotoxic, nephrotoxic, and cytotoxic properties, and despite the routine use of substances with genotoxic potential, such as disinfectants, sterilising agents, drugs, and volatile anesthetics, evidence on the magnitude and characteristics of these exposures remains limited and inconsistent.

Given the growing concern about occupational exposure in healthcare settings, cytogenetic biomonitoring is a valuable tool for detecting early biological effects and supporting preventive occupational health strategies. This study aimed to evaluate the frequency of micronuclei and nuclear abnormalities in exfoliated buccal cells from nurses and dentists to identify potential genotoxic effects associated with chronic occupational exposure in healthcare environments.

However, cytogenetic alterations detected in buccal epithelial cells may be influenced by multiple interacting factors, including occupational exposures, psychosocial stress, lifestyle habits, and individual susceptibility [25]. Therefore, findings derived from cross-sectional biomonitoring studies should be interpreted as associative rather than causal.

## 2. Materials and Methods

### 2.1. Study Design and Population

An exploratory, descriptive, and comparative cross-sectional design was applied. Blood sample collection and participant recruitment were conducted during 2021. A total of 152 volunteers were recruited, comprising 45 nurses, 54 dentists (exposed group), and 53 teachers (control group). All participants were recruited in Toluca, State of Mexico, Mexico, an urban area located in central Mexico. Participants were selected based on predefined inclusion and exclusion criteria. Although the study did not intentionally restrict participation by sex, the final sample consisted only of female participants because the workforce in the participating clinical unit is overwhelmingly female. As a result, all eligible and consenting workers during the recruitment period were women. This reflects the actual demographic structure of the target population rather than a predetermined exclusion criterion.

Exclusion criteria included: exposure to diagnostic X-rays within the preceding 3 months, current or recent pharmacological treatment, and presence of infectious, metabolic, or neoplastic diseases (Table 1).

After applying these criteria, 32 nurses, 41 dentists, and 47 control subjects (teachers) were included in the final analysis. Demographic and exposure-related data were collected via a structured questionnaire, which included variables such as age, sex, smoking status, dietary habits, use of therapeutic drugs, and, for dentists, the number of dental amalgam restorations performed. Nurses were selected from a maternal and perinatal healthcare facility, working across several clinical areas commonly involved in the care of pregnant women, newborns, and postpartum patients, including Obstetrics, Gynecology, Labour and Delivery, Postpartum Care, Neonatology, Neonatal Intensive Care Units, and Maternal–Neonatal Emergency Services. All eligible nurses working in these departments during the data collection period were invited to participate through in-person briefings, institutional email notifications, and posters displayed in staff common areas. Participation was voluntary, and informed consent was obtained from all respondents. A total of 40 eligible nurses were invited; 32 agreed to participate, while eight declined or did not respond.

This exploratory study did not include direct environmental monitoring, internal biomarkers of occupational exposure, or quantitative assessments of psychosocial stress, income level, or workload. Instead, occupational exposure was inferred using professional category as a proxy, an approach commonly applied in early-effect human biomonitoring studies [26]. While suitable for exploratory research, this strategy limits causal interpretation of the observed associations.

### 2.2. Ethical Considerations

The study was approved by the Research Ethics Committee of the “Mónica Pretelini Sáenz” Hospital in Toluca, State of Mexico (approval code: CONBIOETICA-15-CEI-005-20170615). All participants provided written informed consent before inclusion in the study, in accordance with the ethical principles outlined in the Declaration of Helsinki.

### 2.3. Sample Collection

Exfoliated buccal epithelial cells were obtained by gently scraping the inner surfaces of both cheeks with sterile plastic spatulas, after rinsing the mouth with bottled water to remove oral debris. The collected cells were immediately smeared onto two pre-cleaned and labelled microscope slides per subject. The cellular smears were fixed in a freshly prepared methanol–glacial acetic acid (3:1, *v*/*v*; Merck) solution and stored at room temperature until further processing [1].

### 2.4. Staining Procedure

Slides were rehydrated in distilled water for 2 min, then sequentially immersed in 50% and 20% ethanol. Hydrolysis was performed by incubating the slides in 5 M hydrochloric acid (HCl) for 30 min, followed by thorough rinsing with running tap water for 3 min.

Staining was performed with Schiff’s reagent (109033, Merck, Darmstadt, Germany) for 90 min in the dark. Afterwards, slides were rinsed with tap water for 5 min, followed by Milli-Q^®^ water (Merck high-flow system). To enhance contrast, slides were immersed in a 0.2% aqueous solution of brilliant green (L5382, Sigma-Aldrich, St. Louis, MO, USA) for 30 s, rinsed again with Milli-Q water, and air-dried at room temperature [27].

### 2.5. Cytological Analysis

Slides were examined under a Carl Zeiss Axiolab A1 binocular microscope using a 40× objective. A total of 3000 exfoliated epithelial cells per subject (1500 cells per slide) were evaluated to determine the frequency of micronuclei (MN) and other nuclear abnormalities (NAs) according to standardized criteria as described by Tolbert et al. [27] and further refined by Thomas et al. [1].

The nuclear anomalies assessed included: BN, KR, KL, CC, PYK, LN.

Frequencies were expressed as the number of MN and NA per 1000 cells (‰).

### 2.6. Statistical Analysis

#### 2.6.1. Univariate Statistical Analysis

All statistical analyses were performed using R version 4.4.2 [R Core Team, 2024] [28]. Data normality was assessed using the Shapiro–Wilk test. A Kruskal–Wallis multiple-comparisons analysis was performed, followed by pairwise Wilcoxon rank-sum (Mann–Whitney U) tests, adjusted using the Benjamini–Hochberg method.

#### 2.6.2. Multivariate Statistical Analysis

A perMANOVA based on a Euclidean distance matrix and 4999 permutations was conducted using the vegan package (v2.7-2) [29]. Post hoc contrasts were assessed using pairwise permutational MANOVA tests (pairwise.perm.manova) in RVAideMemoire (v0.9.83.12) [30], with Benjamini–Hochberg correction for multiple testing.

To determine overall differences in the profile of oral mucosal cell abnormalities among the evaluated groups, a Multivariate Analysis of Variance (MANOVA) was applied, incorporating the seven dependent variables: BN, KR, KL, CC, PYK, LN and MN. This approach allowed analysis of the multivariate structure of the data and consideration of the correlation among the dependent variables.

The independent factor included three occupational groups: nurses, dentists, and teachers. Before the analysis, the statistical assumptions of multivariate normality, homogeneity of covariance matrices, and absence of extreme collinearity were verified. A significance level of *p* < 0.05 was used.

When the MANOVA was significant, post hoc pairwise comparisons were performed between groups for the multivariate effect, followed by comparisons for each individual variable. All statistical analyses were performed using R version 4.4.2 [28]. Data normality was assessed using the Shapiro–Wilk test. A Kruskal–Wallis multiple comparisons analysis was performed, followed by paired Wilcoxon tests adjusted using the Benjamini–Hochberg method. A multivariate analysis of variance (MANOVA) was performed using the “RVAideMemoire” package (v0.9.83.12) [30].

#### 2.6.3. Principal Component Analysis (PCA)

Principal Component Analysis (PCA) was performed to explore the multivariate structure of the data and evaluate clustering patterns among the three occupational groups based on the seven variables of the cytological assay (BN, KR, KL, CC, PYK, LN, MN).

The PCA was based on the standardised correlation matrix, and the principal components were retained based on their percentage contributions to the total variability. Individuals were plotted along the first two dimensions, and scatter ellipses were added for each group to visually depict each group’s relative positions and extents.

The ellipses are descriptive, not inferential; therefore, statistical significance was obtained exclusively through MANOVAs and permutational MANOVAs (4999 permutations, Pillai’s test). PCA was incorporated as a complementary technique to visualise the patterns found in the inferential multivariate analyses.

## 3. Results

**Figure 1:**
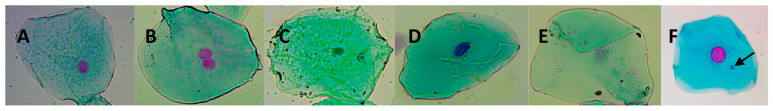
Representative images of nuclear abnormalities identified in exfoliated buccal cells: (**A**) normal nucleus, (**B**) binucleated cell, (**C**) karyolysis, (**D**) karyorrhectic cell, (**E**) condensed chromatin, and (**F**) cell containing one micronucleus (MN). Pictures were taken under the microscope. This staining technique makes the nucleus and MN visible in pink, while the cytoplasm should be greenish, sometimes barely visible, but enough to delineate the cytoplasmic border and detect possible MN within it. It is essential to distinguish MN from the usually present bacteria in the cytoplasm (glued on the surface of the buccal cell), and keratin-rich dots (bodies) that sometimes can appear in the cytoplasm, and without specific DNA staining, could be detected as false MN. Also, due to differences in individual microflora, even after extensive cell washing before fixation and staining, the samples can appear less clean (rich in bacteria and destroyed cell parts), as shown in the pictures, but this does not affect the detection of the nucleus and MN.

A total of 120 female participants were included in the final analysis, comprising 73 healthcare professionals (32 nurses and 41 dentists) and 47 educators (teachers) serving as a non-exposed control group. Representative images of the nuclear abnormalities identified in exfoliated buccal cells are presented in Figure 1.

All participants included in the final analysis were female. This sex homogeneity eliminates sex-related variability in micronucleus frequency, which has been reported mainly for lymphocytes but is less consistent or negligible in exfoliated buccal cells, where some studies have found no significant sex differences [31]. Therefore, sex was not considered a confounding variable in the present analyses. Age was considered the primary demographic variable potentially influencing micronuclei frequency and was evaluated across occupational groups.

### 3.1. Demographic and Occupational Characteristics

Group 1 (nurses) included 32 nurses employed at a tertiary care hospital, with ages ranging from 21 to 70 years and a mean age of 35.88 years. Professional experience ranged from 1 to 38 years, with an average of 10.94 years. The distribution of years of experience was as follows: 1–5 years (*n* = 13); 6–10 years (*n* = 8), 11–20 years (*n* = 7), 21–30 years (*n* = 1), and >30 years (*n* = 3).

Group 2 (dentists) comprised 41 female dentists from private dental practices, aged 23 to 71 years, with a mean age of 36.78. Professional experience ranged from 1 to 34 years, with an average of 9.51 years. The distribution of years of experience was categorized thus: 1–5 years (*n* = 21); 6–10 years (*n* = 7), 11–20 years (*n* = 7), 21–30 years (*n* = 2), and >30 years (*n* = 4).

Group 3 (teachers—control) consisted of 47 female teachers from various educational levels, aged 22–64 years, with a mean age of 39.18. Experience levels were ranged from 1 to 42 years and a mean of 14.52 years and distributed as follows: 1–5 years (*n* = 14); 6–10 years (*n* = 5), 11–20 years (*n* = 15), 21–30 years (*n* = 8), and >30 years (*n* = 5).

Mean age did not differ significantly among the three occupational groups (Kruskal–Wallis test, *p* > 0.05), indicating a comparable age distribution across groups. Consequently, age-related variability is unlikely to account for the observed differences in cytological outcomes.

### 3.2. Nuclear Abnormalities and Micronuclei Frequencies in Buccal Mucosa Cells

A descriptive analysis of nuclear abnormalities and micronuclei frequencies in buccal mucosa cells across the three professional groups is presented in Table 2. Nurses showed the highest mean values for BN, KR, and MN, while dentists and teachers exhibited comparatively lower frequencies of these alterations. Teachers showed the lowest levels of KL CC, LN, and. Overall, notable variability was observed within each group, as reflected by the standard deviations, particularly among nurses. These findings suggest profession-related differences in the distribution of nuclear abnormalities.

### 3.3. Comparison of MN and NA Frequencies Among Groups

#### 3.3.1. Univariate Comparisons

Due to the non-Gaussian distribution of the data, post hoc pairwise comparisons between independent groups were performed using the Wilcoxon rank-sum statistic (Mann–Whitney U) with Benjamini–Hochberg correction. Univariate comparisons for each of the seven variables are summarised in Table 3. Overall, the most consistent differences were observed in KR frequency, with the nurses’ group showing significantly lower values than the dentists’ (*p* < 0.001) and the teachers’ (*p* < 0.001). MN frequency also revealed differences between nurses and teachers (*p* = 0.00497) but not between nurses and dentists. The remaining variables (BN, KL, CC, PYK, LN) did not show significant differences between the groups, as indicated by the *p*-values.

The results indicate that the differences between groups are mainly influenced by variations in KR and, to a lesser extent, in MN. At the same time, the other variables did not contribute significantly to the observed contrasts.

#### 3.3.2. Multivariate Analysis

The individual results of the oral mucosa cell abnormality assay are presented in Table 4. MANOVA analysis showed statistically significant multivariate differences between groups (*p* = 0.003 for nurses vs. dentists; *p* = 0.003 for nurses vs. teachers), indicating a distinct pattern of cell abnormalities in the nurses group compared to the other two groups.

To complement the MANOVA findings, multivariate significance between groups was also evaluated using a perMANOVA based on a Euclidean distance matrix with 4999 permutations. Pairwise post hoc comparisons were conducted with permutational tests and *p*-values were adjusted using the Benjamini–Hochberg method. Consistent with the overall multivariate results, nurses vs. dentists and nurses vs. teachers showed significant differences, whereas dentists vs. teachers did not. The detailed outcomes of these comparisons are presented in Table 4.

In contrast, the groups of dentists and teachers showed no significant differences at the multivariate level (*p* = 0.486), suggesting similar cytological profiles.

#### 3.3.3. Principal Component Analysis (PCA)

PCA enabled us to represent the variability among the seven cytological variables in a two-dimensional space. Principal Component 1 (PC1) explained 36% of the total variability, while PC2 explained 19.6%, so that both components captured 56% of the combined variation.

**Figure 2:**
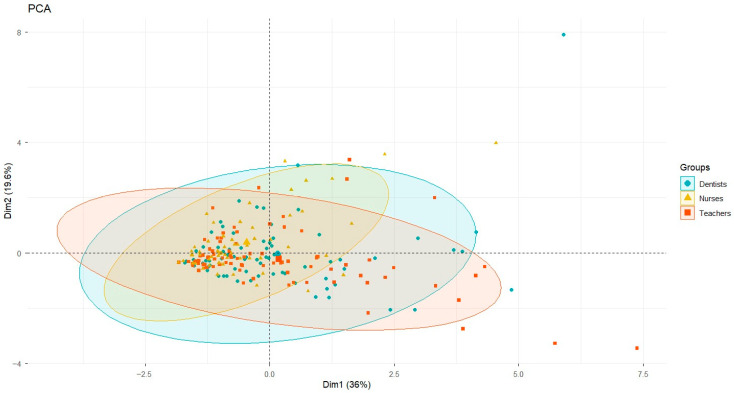
Principal Component Analysis (PCA) of the seven buccal mucosa cytogenetic/cytotoxicity variables (BN, KR, KL, CC, PYK, LN, MN). The first two components explain 56% of the total variance (PC1 = 36%, PC2 = 19.6%). Each point represents an individual participant, and ellipses indicate the dispersion and centroid location for each occupational group (Nurses—yellow color, Dentists—blue color, Teachers—red color). The PCA suggests a distinct multivariate profile for the nurses’ group, consistent with the significant differences detected by the MANOVA and permutation MANOVAs. Ellipses are descriptive and do not indicate inferential significance.

In the bivariate plot (Figure 2), each point corresponds to an individual, and different symbols distinguish between the groups. The ellipses surrounding each cluster represent the internal dispersion and the average position of each group.

The visual pattern showed a clear separation of the nurses’ group, whose clustering shifted relative to that of dentists and teachers, suggesting a distinct cytological profile. In contrast, dentists and teachers showed greater overlap, consistent with their multivariate statistical similarity.

To facilitate interpretation of the PCA, the loadings of each cytological variable on the first two principal components were examined (Supplementary Table S1). PC1 explained 36.0% of the total variance and was strongly driven by high positive loadings of condensed chromatin (CC; loading = 0.86), karyorrhexis (KR; loading = 0.76), binucleated cells (BN; loading = 0.75), and pyknotic nucleus (PYK; loading = 0.59). These biomarkers are commonly associated with cytotoxicity, nuclear fragmentation, and genomic instability, indicating that PC1 primarily reflects a cytotoxic/genotoxic response dimension.

PC2 accounted for an additional 19.6% of the variance and was dominated by micronuclei (MN; loading = 0.72) and lobed nucleus (NL; loading = 0.64), whereas KR and PYK loaded negatively on this component (−0.36 and −0.41, respectively). This pattern suggests that PC2 captures a partially opposing axis of nuclear alterations, distinguishing samples with higher frequencies of MN and NL from those characterised by increased nuclear degeneration markers.

The displacement of the nurses’ group along PC1, therefore, reflects a greater contribution of biomarkers indicative of cytotoxic stress and genomic instability, providing a biological basis for the distinct multivariate profile observed in this occupational group.

Age and years of professional experience were not included as active or supplementary variables in the PCA. Preliminary Spearman correlation analyses showed weak and mostly negative correlations between these demographic variables and the cytogenetic biomarkers. In addition, mean ages were comparable across the three occupational groups, reducing the likelihood that the observed multivariate separation was primarily driven by age rather than occupational factors.

#### 3.3.4. Complementarity with MANOVA

PCA suggested structural differences in the multivariate pattern, which were numerically confirmed by the inferential analyses. Permutational comparisons (4999 permutations, Pillai test) showed that in nurses vs. dentists, *p* = 0.0045; nurses vs. teachers, *p* = 0.0045; and teachers vs. dentists, *p* = 0.498. These results indicate that the nurses’ group shows a significantly different pattern of cellular abnormalities compared with the other two groups. At the same time, dentists and teachers do not differ from each other, a pattern that coincides with the overlap observed in the PCA.

## 4. Discussion

The findings of this study indicate that maternal–perinatal services concentrate a constellation of occupational risks whose combined and cumulative effects may negatively impact the health of nursing staff. In these highly specialised obstetric and neonatal environments, workers are routinely exposed to complex mixtures of biological, physical, and chemical agents, including substances with recognised genotoxic potential such as mercury [15]. Although the use of mercury-containing devices has decreased, their residual presence, whether from older instruments or secondary environmental contamination, may still represent a relevant source of exposure capable of inducing DNA damage even at low concentrations. Mercury, a highly toxic heavy metal that exists in elemental, inorganic, and organic forms, is known to cause neurotoxic, nephrotoxic, immunotoxic, and genetic effects [17,32,33]. In maternal–perinatal settings, where most workers are women of reproductive age, these risks are particularly concerning due to their potential implications for fertility, pregnancy outcomes, and long-term health.

As a cross-sectional design, this study identifies associations but cannot establish causal relationships between occupational category and cytogenetic profiles [34,35]. Biomonitoring endpoints such as micronucleus frequency reflect early biological effects potentially linked to exposure, but they do not constitute definitive evidence of cause-and-effect relationships [25].

The BMCyt assay is a well-validated, minimally invasive technique for detecting early chromosomal damage and genomic instability in human populations. It has been widely applied in occupational biomonitoring studies to identify subtle biological effects associated with low-dose and mixed exposures to genotoxic agents [1,25]. However, the frequency of micronuclei and other nuclear abnormalities may also be modulated by non-chemical factors, such as psychosocial stress, nutritional status, smoking, age, and other lifestyle characteristics [13]. These variables were not quantitatively assessed in the present study, and their potential confounding influence cannot be excluded.

In this context, evaluating MN and NA in buccal epithelial cells provides a sensitive approach to assess genotoxicity and cytotoxicity among healthcare professionals. Consistent with the literature, the increased presence of MN and NA observed in nurses compared with the dentists and non-exposed control group of teachers underscores the relevance of occupational exposure to genotoxic agents in clinical environments. These results highlight the need to strengthen preventive measures and monitoring strategies in maternal–perinatal facilities, where multidimensional occupational hazards may converge, contributing to cumulative genetic and cytotoxic effects over time.

The results indicate a significantly higher frequency of MN among nurses than among dentists and teachers, suggesting occupational exposure to genotoxic agents. Nurses are commonly exposed to anesthetic gases, disinfectants, cytotoxic drugs, and, historically, mercury-containing thermometers and sphygmomanometers, many of which are known to cause DNA damage [21,22]. Furthermore, psychosocial stressors such as prolonged shifts, emotional fatigue, and burnout may contribute to genomic instability through oxidative stress and impaired DNA repair mechanisms.

MN frequencies among dentists did not differ from those in the control group. Dental professionals may be exposed to mercury vapours from amalgam preparation, volatile anesthetics, and other dental materials, often in poorly ventilated environments. Diet, ergonomic stress, and possible co-exposures in clinical practice may also modulate individual susceptibility to genotoxic damage [21]. However, the absence of a statistically significant increase in NA or MN among dental staff compared with teachers suggests either lower exposure or more effective protective measures in dental settings.

Teachers, selected as a control group due to their lack of occupational exposure to known genotoxic agents, had the lowest MN frequencies, confirming their suitability as a baseline reference population. Their inclusion strengthens the comparative framework of this study.

The multivariate analysis revealed distinct differences in the combined profile of buccal mucosa cell abnormalities, with nurses exhibiting a significantly different cytological pattern compared with dentists and teachers. This finding suggests that nursing staff may experience a unique constellation of occupational exposures that can induce early genotoxic and cytotoxic effects. Similar patterns have been reported in previous studies showing elevated frequencies of micronuclei, nuclear abnormalities or primary DNA damage in nurses chronically exposed to disinfectants, volatile chemicals, and complex mixtures of hospital pollutants [36,37,38].

Among the evaluated biomarkers, KR showed the most consistent differences, highlighting its sensitivity for detecting cytotoxic responses in low-dose exposure scenarios. Micronucleus (MN) frequency was also significantly higher in nurses compared with dentists and teachers, reinforcing its value as a robust biomarker of DNA damage and genomic instability [39,40]. Comparable increases in MN frequencies have been documented among nurses handling sterilising agents, anesthetic gases, and cleaning products, exposures widespread where chemical use is recurrent and ventilation may be limited [41,42,43,44].

In contrast, the absence of significant differences between dentists and teachers suggests that not all healthcare professionals or occupational groups with potential chemical exposure exhibit comparable levels of genotoxicity, likely reflecting variability in exposure intensity, type of substances handled, and adherence to protective practices, among other factors [45,46].

Principal component analysis provided a clear visual representation of the data’s multivariate structure, confirming the trends identified by MANOVA. The separation of the nurses’ group along the first two principal components, which together explain 56% of the total variability, indicates a distinctive cytological profile within this population.

The biological interpretation of these components was based on the magnitude and direction of the variable loadings, which quantify each biomarker’s contribution to the multivariate structure of the data, following standard principles of principal component analysis [47].

Importantly, the PCA interpretation was supported by the variable loadings on the first two principal components. PC1, which accounted for the largest proportion of variance (36.0%), was primarily driven by biomarkers associated with cytotoxicity and genomic instability, including condensed chromatin, karyorrhexis, binucleated cells, and pyknotic nuclei. The coordinated contribution of these alterations suggests an underlying pattern of cellular stress, nuclear damage, and impaired cell division.

PC2 (19.6% of variance) was mainly influenced by micronuclei and lobed nuclei, while markers such as karyorrhexis and pyknotic nuclei showed negative loadings. This structure indicates that PC2 reflects an alternative pattern of nuclear alterations, partially opposing classical degenerative changes and highlighting that different types of nuclear damage do not necessarily co-occur within the same individuals.

This loading structure confirms that the multivariate separation observed in the PCA is not arbitrary but rather, reflects a coordinated increase in biologically meaningful biomarkers consistent with chronic exposure to complex occupational stressors in maternal–perinatal healthcare environments.

In contrast, the overlap between dentists and teachers is consistent with the absence of significant differences in the multivariate analysis. Overall, the concordance between the descriptive patterns revealed by PCA and the inferential results from MANOVA supports the interpretation that nursing staff exhibit a distinct pattern of cellular abnormalities, potentially associated with specific occupational exposures, factors related to the hospital environment, or even behavioural determinants, such as dietary patterns in the workplace. This methodological alignment enhances the robustness of the analysis and supports a more comprehensive understanding of the multivariate behaviour of the biomarkers under evaluation.

The combined use of principal component analysis (PCA) and multivariate analysis of variance (MANOVA) enhances the reliability of findings. PCA offers a clear visualisation of the data structure by extracting linear combinations (principal components) that explain most of the variability [48], and MANOVA provides formal inference regarding group differences [49]. The strong correspondence between the clustering of nursing samples in the PCA scatterplot and the significant differences detected by MANOVA supports the conclusion of a characteristic cytological profile in nurses. This integrative approach, commonly used in genomics and high-dimensional biomedical contexts [50], increases confidence that the observed separation is genuine and driven by biological or occupational factors associated with nursing practice in high-exposure clinical settings.

These results support the utility of the buccal cytome assay as a sensitive biomonitoring tool for detecting early genotoxic and cytotoxic effects in healthcare workers and highlight the need to strengthen occupational hygiene measures across clinical units, particularly in nursing staff operating in high-demand environments such as maternal–perinatal services.

The findings align with previous literature supporting the use of the BMCyt assay as a reliable biomarker for biomonitoring populations at risk of chronic low-dose exposure to genotoxic agents [1,6,13]. This assay is particularly valuable in occupational health due to its non-invasive nature, low cost, and sensitivity in detecting early DNA damage.

This analysis highlights the need for specific studies that quantify actual exposures in these settings and enable the development of evidence-based interventions to protect nursing staff and improve the quality of maternal and perinatal care.

The study presents several notable strengths that reinforce the relevance of its findings. The combined use of MANOVA and PCA enabled a comprehensive evaluation of both the statistical significance and the multivariate structure of the biomarker data, providing a more nuanced interpretation of group differences. The incorporation of permutational tests reduced reliance on strict normality assumptions, increasing analytical robustness. Moreover, the selection of widely validated biomarkers for human biomonitoring enhanced the credibility and translational value of the results.

Despite these strengths, certain limitations warrant consideration. The cross-sectional design and the absence of direct exposure measurements limit causal inference, and key confounding variables, including nutritional status, lifestyle habits, and DNA repair-related genetic polymorphisms, were not fully controlled. The relatively small sample size, particularly in subgroups stratified by years of professional experience, may also limit statistical power and the generalizability of the findings. In addition, non-chemical factors such as psychosocial stress, shift work, workload, recovery time, and broader lifestyle characteristics can independently influence oxidative stress, genomic stability, and micronucleus frequency. These variables were not quantitatively assessed in the present study, and their potential confounding effects cannot be entirely excluded.

Future research should prioritise longitudinal designs, larger and more diverse cohorts, and the incorporation of molecular assays (e.g., the comet assay and γ-H2AX foci) to refine the assessment of genomic instability. A more detailed characterisation of occupational exposures, including environmental monitoring and biomonitoring of internal doses, would strengthen causal interpretations. Additionally, integrating psychosocial and ergonomic stressors into exposure frameworks may provide a more holistic understanding of genomic instability in healthcare settings and further illuminate the mechanisms underlying the cytogenetic alterations observed in this study.

Future studies should incorporate longitudinal designs and direct exposure assessments, including environmental monitoring and internal dose biomarkers, to clarify the temporal relationship between occupational exposures and cytogenetic endpoints [25,51].

## 5. Conclusions

This study identifies a distinct cytogenetic profile in nurses compared with dentists and teachers, as assessed by the BMCyt assay, suggesting an association between nursing work environments and early genotoxic or cytotoxic biomarkers. Given the increasing demand for healthcare systems and the diverse exposures encountered in clinical settings, it is essential to implement preventive measures and regular biomonitoring protocols to mitigate long-term health risks. Further longitudinal research (with larger cohorts and complementary genotoxicity assays) incorporating direct exposure measurements, psychosocial factors, and a broader assessment of potential confounders is required to clarify causal pathways, characterise these occupational hazards better, and support effective preventive occupational-health strategies and evidence-based policy development.

## Figures and Tables

**Table 1:** Inclusion and exclusion criteria and participant flow by occupational group.

Category	Description
Study design	exploratory, descriptive, comparative cross-sectional study
Study population	nurses, dentists, and teachers employed during the recruitment period
Inclusion criteria	active employment in the corresponding occupational group during the study period; willingness to participate and provision of written informed consent;
Exclusion criteria	exposure to diagnostic X-rays within the previous 3 months; current or recent pharmacological treatment; presence of infectious, metabolic, or neoplastic diseases
Volunteers initially recruited (*n*)	nurses: 45; dentists: 54; teachers (control group): 53
Participants excluded after screening (*n*)	nurses: 13; dentists: 13; teachers: 6
Final sample included in analysis (*n*)	nurses: 32; dentists: 41; teachers: 47
Total final sample (*n*)	120

Note: All participants were female, reflecting the workforce demographics at the participating institutions; sex was not used as an a priori exclusion criterion.

**Table 2:** Mean and standard deviation of micronuclei (MN) and nuclear abnormalities by profession in buccal mucosa cells.

Profession	N	BN	KR	KL	CC	PYK	LN	MN
nurses	32	130.3 ± 183.0	49.1 ± 19.1	3.6 ± 4.3	5.8 ± 5.6	1.6 ± 1.6	0.71 ± 0.31	12.3 ± 22.6
dentists	41	118.7 ± 69.7	16.8 ± 16.6	3.6 ± 4.3	4.7 ± 4.6	1.7 ± 1.8	0.2 ± 0.5	3.6 ± 4.3
teachers—control	47	118.6 ± 79.3	18 ± 27	0.5 ± 1.0	5.5 ± 8.3	1.6 ± 2.5	0.079 ± 0.19	2.2 ± 3.7

BN: binucleated cells; KR: karyorrhexis; KL: karyolysis; CC: condensed chromatin; PYK: pyknotic nucleus; LN: lobed nucleus; MN: micronuclei. The values represent the averages ± standard deviation.

**Table 3:** Univariate statistical comparisons for the seven buccal cytome assay variables.

Group	The Group Compared with	BN	KR	KL	CC	PYK	LN	MN
dentist	nurse	0.371	**˂0.001**	0.597	0.268	0.212	0.687	0.128
teacher	nurse	0.132	**˂0.001**	0.597	0.268	0.212	0.981	**0.00497**
teacher	dentist	0.371	0.638	0.625	0.951	0.815	0.687	0.128

BN: binucleated cells; KR: karyorrhexis; KL: karyolysis; CC: condensed chromatin; PYK: pyknotic nucleus; LN: lobed nucleus; MN: micronuclei. The values represent statistical differences (*p*).

**Table 4:** Multivariate comparison of buccal mucosa cell abnormalities among study groups.

Group	Group Compared	*p* Value
dentists	nurses	**0.003**
teachers	nurses	**0.003**
teachers	dentists	0.486

## Data Availability

The original contributions presented in this study are included in the article/Supplementary Materials. Further inquiries can be directed to the corresponding authors.

## Supplementary Materials

The following supporting information can be downloaded at: https://www.mdpi.com/article/10.3390/toxics14010061/s1, Table S1. Loadings of the seven buccal cytome assay biomarkers on the first two principal components (PC1 and PC2) obtained from principal component analysis (PCA).

## Abbreviations

BMCyt	Buccal micronucleus cytome assay
PCA	Principal Component Analysis
PC1	Principal Component 1
PC2	Principal Component 2
MN	Micronuclei
NA	Nuclear abnormalities
IARC	International Agency for Research on Cancer
BN	Binucleated cells
KR	Karyorrhexis
CC	Condensed chromatin
NL	Lobed nucleus
PYK	Pyknotic nucleus
KL	Karyolysis
