# Supplementary material for: Micronuclei and Nuclear Abnormalities in Oral Mucosa as Indicators of Genotoxicity in Healthcare Professionals
Source: Toxics. 2026 Jan 8;14(1):61. doi: 10.3390/toxics14010061 (PMC12846128; doi:10.3390/toxics14010061)
Supplement: Supplementary file 1 [file toxics-14-00061-s001.zip › toxics-4043271-supplementary.pdf]

toxics

Article

Micronuclei and Nuclear Abnormalities in Oral Mucosa as Indicators of Genotoxicity in Healthcare Professionals

Juana Sánchez-Alarcón [1,2], Stefano Bonassi [3,4,*], Mirta Milić [5], Ninfa Ramírez-Durán [6], Keila Isaac-Olivé [7] and Rafael Valencia-Quintana [2,*]

1 Doctorado en Ciencias de la Salud, Facultad de Ciencias de la Conducta, Universidad Autónoma del Estado de México, Toluca 50180, Estado de México, Mexico; juana.sanchez@uatx.mx
2 Cuerpo Académico Ambiente y Genética UATLX-CA-223, Laboratorio "Rafael Villalobos-Pietrini" de Toxicología Genómica y Química Ambiental, Facultad de Agrobiología, Universidad Autónoma de Tlaxcala, Ixtacuixtla 90120, Tlaxcala, Mexico
3 Department of Human Sciences and Quality of Life Promotion, San Raffaele University, 00166 Rome, Italy
4 Unit of Clinical and Molecular Epidemiology IRCCS San Raffaele Pisana, 00166 Rome, Italy
5 Division of Toxicology, Institute for Medical Research and Occupational Health, 10000 Zagreb, Croatia; mmilic@imi.hr
6 Laboratorio de Microbiología Médica y Ambiental, Facultad de Medicina, Universidad Autónoma del Estado de México, Toluca 50180, Estado de México, Mexico; nramirezd@uaemex.mx
7 Laboratorio de Investigación en Teranóstica, Facultad de Medicina, Universidad Autónoma del Estado de México, Toluca 50180, Estado de México, Mexico; kisaaco@uaemex.mx
* Correspondence: stefano.bonassi@sanraffaele.it (S.B.); prvq2004@yahoo.com.mx (R.V.-Q.)

Table S1. Loadings of the seven buccal cytome assay biomarkers on the first two principal components (PC1 and PC2) obtained from principal component analysis (PCA).

Variable	PC1	PC2
Condensed chromatin (CC)	0.86	−0.10
Karyorrhexis (CR)	0.76	−0.36
Binucleated cells (BN)	0.75	0.35
Pyknotic nucleus (NP)	0.59	−0.41
Karyolysis (KL)	0.40	−0.05
Lobed nucleus (NL)	0.29	0.64
Micronuclei (MN)	0.24	0.72

The loadings of the seven cytogenetic biomarkers on PC1 and PC2 are reported in Table S1. Age and years of professional experience were not included as active or supplementary variables in the PCA. Preliminary Spearman correlation analyses showed weak and mostly negative correlations between these demographic variables and the cytogenetic biomarkers. In addition, mean ages were comparable across the three occupational groups, reducing the likelihood that the observed multivariate separation was primarily driven by age rather than occupational factors.
